# SInC: an accurate and fast error-model based simulator for SNPs, Indels and CNVs coupled with a read generator for short-read sequence data

**DOI:** 10.1186/1471-2105-15-40

**Published:** 2014-02-05

**Authors:** Swetansu Pattnaik, Saurabh Gupta, Arjun A Rao, Binay Panda

**Affiliations:** 1 Ganit Labs, Bio-IT Centre, Institute of Bioinformatics and Applied Biotechnology, Biotech Park, Electronic City Phase I, Bangalore 560100, India; 2 Strand Life Sciences, Kirloskar Business Park, Bellary Road, Hebbal, Bangalore 560024, India

## Abstract

**Background:**

The rapid advancements in the field of genome sequencing are aiding our understanding on many biological systems. In the last five years, computational biologists and bioinformatics specialists have come up with newer, better and more efficient tools towards the discovery, analysis and interpretation of different genomic variants from high-throughput sequencing data. Availability of reliable simulated dataset is essential and is the first step towards testing any newly developed analytical tools for variant discovery. Although there are tools currently available that can simulate variants, none present the possibility of simulating all the three major types of variations (Single Nucleotide Polymorphisms, Insertions and Deletions and Copy Number Variations) and can generate reads taking a realistic error-model into consideration. Therefore, an efficient simulator and read generator is needed that can simulate variants taking the error rates of true biological samples into consideration.

**Results:**

We report SInC (**S**np, **In**del and **C**nv) an open-source variant simulator and read generator capable of simulating all the three common types of biological variants taking into account a distribution of base quality score from a most commonly used next-generation sequencing instrument from Illumina. SInC is capable of generating single- and paired-end reads with user-defined insert size and with high efficiency compared to the other existing tools. SInC, due to its multi-threaded capability during read generation, has a low time footprint. SInC is currently optimised to work in limited infrastructure setup and can efficiently exploit the commonly used quad-core desktop architecture to simulate short sequence reads with deep coverage for large genomes.

**Conclusions:**

We have come up with a user-friendly multi-variant simulator and read-generator tools called SInC. SInC can be downloaded from http://sourceforge.net/projects/sincsimulator.

## Background

The rapid advancements in the field of genome sequencing is aiding our understanding of genome organisation in many biological systems [1-3]. These tools are intended to analyse high throughput next-generation sequence (NGS) data and present biologically relevant interpretations. Given the high throughput nature of present day genomics, heuristic algorithms are implicated to identify or predict genome variations as small as single base nucleotide substitutions (SNVs) to insertion-deletion events (indels) and copy number variations (CNVs). Hence, it is imperative for developers of NGS data analysis pipelines to establish the limits of their predictions based on simulated data as in current practice. In the last five years, computational biologists and bioinformatics specialists have developed new algorithms for different types of variant calling, have implemented existing algorithms for short-read mapping to reference genomes and/or optimized pipelines to perform a specific type of primary and secondary analysis [4-19]. SNVs, indels and CNVs are the most common types of biological variations in the genome. The tools to detect these variants have the common objective of finding novel variations with low frequency of false positives, rediscovering known variations in the genome of interest and facilitate subsequent genome visualization and interpretation. Hence, availability of reliable and realistic simulated dataset bearing the three major types of genomic variations (SNVs, indels and CNVs) is critical to test the operational limitations of newly developed or existing tools. This approach allows computational biologists to generate simulated datasets with biological meaning and sensitive to systematic error inherent to different sequencing technology platforms.

Although, next-generation sequencing (NGS) instruments generate reads of various lengths and with varying error profiles, the most popular source of data remains sequencing instruments from Illumina, which employs a sequencing-by-synthesis [20] chemistry to generate short-reads. Keeping this in mind, we have developed an efficient, fast simulator and a read generator that mimics sequencing quality generated by Illumina platform. Hence, SInC uses a realistic error model based on base quality values of reads generated by the most prevalent sequencing platform, hence catering to the larger interest group. Although we have used the Illumina-derived base quality values, it can easily be adopted for any other sequencing platform by supplying an instrument-specific error profile.

Currently available tools can either generate platform-specific, error-profile based reads or simulate reads across platforms [21-28]. It is also in our interest of disambiguation to classify the existing simulators into two major classes based on their functionality. First, the stand-alone read generators (RG) like Metasim [28], Flowsim [22], 454Sim [24], Pbsim [21], GenFrag [29] and ART [25] among others with functionality limited to read generation. The second class of simulators (SRG) include pIRS [23], GEMsim [26], dwgSIM [30] (based on wgsim of samtools), which have the option of simulating genomic variations coupled with read generation functionality. Each of the above mentioned tools, although has its own set of advantages, suffers from either having a simplistic error model (in the case of GenFrag), errors that does not model real data (in the case of dwgSIM), does not assign quality values to reads (in the case of Metasim), does not simulate Illumina reads (in the case of Flowsim) or does not simulate multiple types of variations (in the case of pIRS and GEMsim). Interestingly, none of the existing SRG simulators present the option to simulate CNVs. Hence, we have developed and implemented a C-program, SInC, to enable simulation of all the three major types of genomic variations, SNVs, indels and CNVs, coupled with a multi-threaded, error-profile based read generator. SInC has obvious advantages over the popular SRG simulators as dwgSIM simulates reads with identical dummy base quality values relieving the data of any base-quality related effects, pIRS cannot simulate CNVs and GEMsim simulates only SNVs. SInC models errors based on real data from Illumina instruments as in pIRS and additionally presents fine tuned options to replicate biologically meaningful variant simulations including CNVs. The multi-threaded algorithm in SInC for read generation provides substantial advantage in run time and allows for seamless simulation of high coverage data in a desktop environment.

Here we present an evaluation of SInC using commonly used SNV, indel and CNV detection tools. The speed, accuracy and efficiency was compared against other popular simulators and read generators.

## Implementation

SInC performs two jobs; first it simulates variants (simulator) and then it generates reads (read generator). SInC simulator consists of three independent modules (one each for SNV, indel and CNV) that can either be executed independently in a mutually exclusive manner or in any combination.

### SNV simulation

The exact frequency of SNPs in the human genome has not yet been determined accurately. Based on inferences from 629 complete genomes representing several human populations in the 1000 genome data, the current range of frequency of SNV lies between one per 300 to 1000 bases [31]. For this purpose, we have assumed that the substitution events in human genome are independent and random. SInC simulator accepts a user defined percentage value to simulate SNVs. The algorithm identifies this percentage value as the fraction of genome to estimate the number of SNVs and simulates SNVs across the genome. To maintain positional identities of these SNVs with respect to their frequency, that are normally distributed over the sequenced genome, the mean distance of separation (*DAvg*) between SNVs is calculated (see Additional file 1 and Additional file 2).

**Figure 1:**
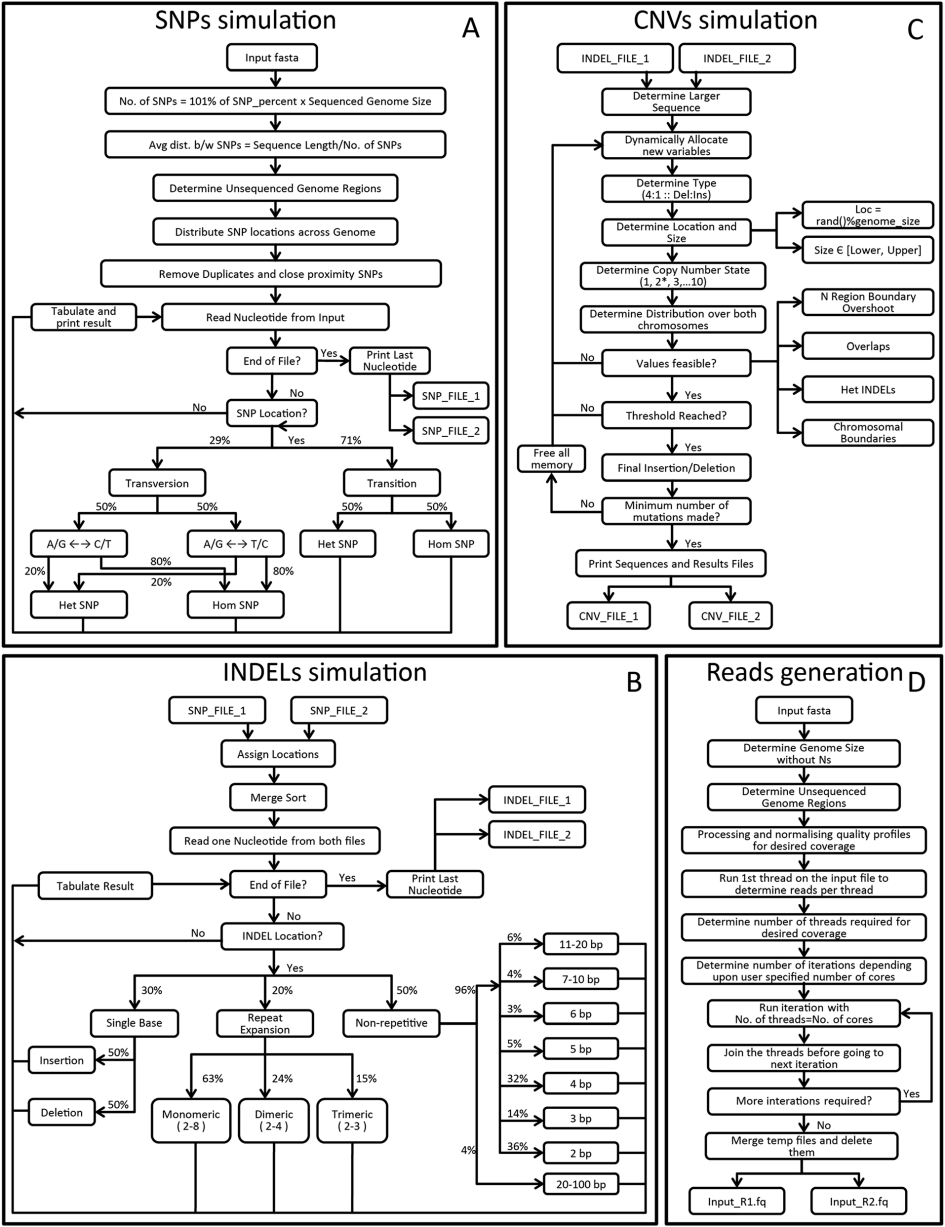
Flowcharts indicate the algorithm implemented in simulation of A) SNP, B) Indel, C) CNV and subsequent D) read generation process.

This ensures that the simulated SNVs are well distributed over the genome. A positional filter is applied to remove the outlier SNVs, which are less than 15 bases apart. SInC simulator neglects SNVs simulated in the N-regions of the genome (where there is no A, T, G or C). Then the algorithm applies a user-defined transition to transversion (Ti/Tv) metric to maintain the biological significance of the SNVs across the genome. A Ti/Tv ratio of 2.1 was maintained across the population of simulated SNVs with 20% inherent heterozygosity to simulate human genome data as previously reported [32]. The flow chart illustrated in Figure 1A depicts the algorithm for simulating SNVs.

### Indel simulation

Insertion and deletion (indel) events have a wide range of size-based variability. SInC simulator simulates short, medium and large indels in the range of 1-10 bp 11-20 bp and 21-100 bp respectively in concordance with earlier studies [33]. The ratio of incidence of insertions to deletions and heterozygous to homozygous indels in human genome is set to 1:1 based on previous observations [33]. The flow chart in Figure 1B depicts the algorithm for simulating indels.

The algorithm first randomly generates the position for indels and then uses a filter to replace any indel within the region of the N-region of the genome (no A, T, G or C assigned) with one in the sequenced region. To remove duplicates, the simulated indels are coordinate sorted and only the unique locations are retained. Usually, a redundancy of 2-5% is filtered out post coordinate sorting (that can result from either duplicates or un-sequenced regions). Hence, an additional 5% of indels are generated at the initial stage of the algorithm to account for the loss of indels at the duplicate removal step. In the next stage of the algorithm, the frequency of short, medium and large indels was factored in based on previous literature evidence for their distribution in human [33]. The indel simulation produces two output files assuming the bi-allelic nature of human genes, each containing allele-specific coordinate information of simulated indels. Among the total number of simulated indels, the algorithm simulates 30% single base indels, 20% repeat expansions, 49% 2-20 bp indels, and 1% long indels including repeat expansions (see Additional file 2 and Additional file 3).

### CNV simulation

The CNV simulation constitutes the final step of the simulation algorithm, as it can ply in a sequential manner post indel simulation. Since the input files from indel simulation may contain heterozygous indels, which may be of unequal size, hence the CNV module takes it into consideration and prevents the possibility of boundary overlaps with indels. The flow chart in Figure 1C depicts the algorithm for simulating CNVs.

Unlike the indel module, here the size and location of the variants are both generated dynamically with the flow of the program after obtaining the feed from the user to determine the number of CNVs and their range of size distribution (upper and lower limit). Such simulated data is particularly useful to test the accuracy and sensitivity of a new or existing CNV caller across a wide range of CNV sizes. The algorithm then filters the simulated CNVs based on its coordinates. First the span of each of the CNVs are evaluated to ensure correspondence with chromosomal boundary in either allele and subsequently the CNV boundaries are checked for overlap with neighboring CNVs. The CNV is logged and the next iteration of location and size are generated upon meeting the aforementioned conditions. Unlike the SNV and indel simulation modules, the annotation data for both alleles in the CNV module is stored in the same file in the form of a tabular data. The tool also outputs a simplified results file (similar to a BED file), which can be read easily by any program for visualizing CNVs.

### Read generation

SInC has a read generator part that generates short reads using a multi-threaded approach utilizing the parallel processing power of commonly used quad-core desktop/laptop architecture. The process of read generation uses a quality profile distribution-based error-model. We have used publicly available 100 bp read pair data derived by using Illumina instrument from the SRA database to generate the quality profile distribution and assessed the quality distribution for both forward and reverse reads of the training set and data post read generation (Additional file 4). For customization purpose, the tool is provided as a standalone tool in the SourceForge package so that the user can generate independent error profiles for reads with certain read lengths to be used during the read generation stage. The read generation algorithm follows a “divide and conquer” approach where each thread spans the input sequence once and the number of reads required to obtain the user defined coverage are pooled from the estimated number of threads. User-specified cores utilization is implemented in the SInC read generator to prevent over-utilization of available CPU resources. The other major user defined parameters, include read length, error profile, insert size (inner distance) and standard deviation of insert size (see Additional file 2). The algorithm initially creates one thread, which generates reads for the input fasta file. Depending on the read pairs generated in the first run, the numbers of threads required to obtain the desired coverage are calculated and then executed in an iterative manner based on the number of cores specified by the user (Figure 1D). SInC is optimized to run with 4 threads suiting a quad-core processor.

### Evaluation of SInC

#### Variant re-discovery

**Figure 2:**
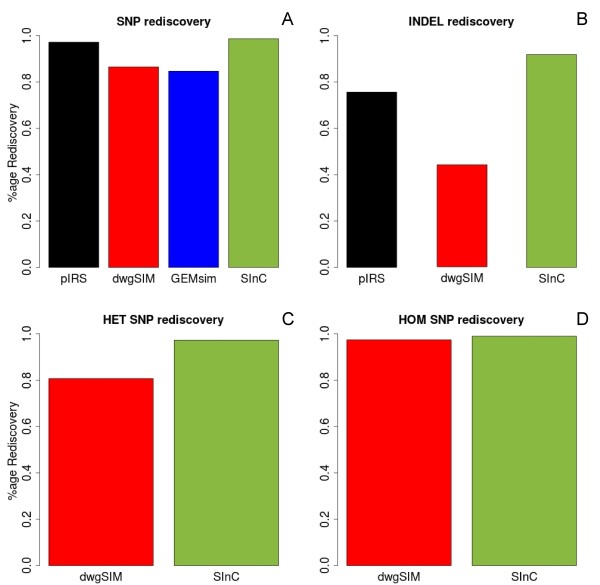
**Variant rediscovery statistics.** Percentages of simulated variants performed using GATK and PINDEL for identification are shown of **A)** SNVs and **B)** indels respectively. The rediscovery of indels based on size specificity was also performed and is given in Additional file 3. The rediscovery percentages of **C)** heterozygous and **D)** homozygous SNVs are compared.

We used human chromosome 22 sequence from the UCSC build hg19 for generating SNVs and indels using all the four different SRG simulators. The SNV rate, indel percentage and coverage was maintained across all the tools and the resulting reads were aligned using Novoalign [14]. These mapped files were subject to SNV and indel detection by GATK [4] and Pindel [6] respectively (see Results, Figure 2). The predicted SNVs and indels from the different simulators were compared to the actual number of incorporated variants to estimate the percentage rediscovery. Rediscovery percentage using Pindel has a limitation that it merges short indels within a span of 40 nucleotides of each other leading to a slight loss (less than 1%) of rediscovered indels across all the simulators (see Additional file 2).

### Time profiling

**Figure 3:**
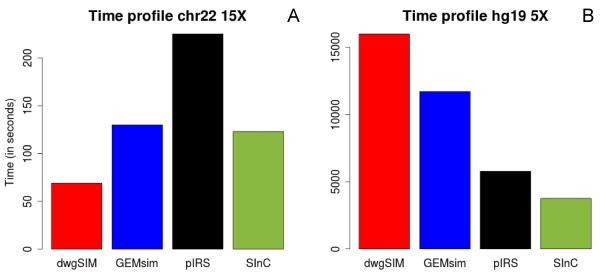
**Time profiles of the different simulators used.** Time elapsed to perform one complete simulation with default options using single core across different simulators. **A)** For chromosome 22 at 15X **B)** For human whole genome (hg19) at 5X.

Given the high-throughput nature of NGS data, generating the bulk of simulated data still remains a time consuming process. Hence, we have implemented a “divide and conquer” approach to the read generation module to reduce the time footprint in generating high coverage data. This property allows user to simulate data at a high coverage (50X – 100X) without inordinate expense of time. SInC can utilize 1 to 4 threads for optimal function. Our comparison was set up based on default use of 1 core ranging upto a maximum utilization of 4 cores in SInC versus the other tools (see Results, Figure 3). Details are provided in the Additional file 2.

### Transition/Transversion (Ti/Tv) ratio

A transition mutation involves a change from purine to purine or pyrimidine to pyrimidine and a tranversion mutation involves a change from pyrimidine to purine or vice versa. This makes a transversion event twice as favourable as a transition event for any random mutation event. Hence, the Ti/Tv ratio for a random variation resulting from systematic errors in the sequencing technology, alignment artifacts and data processing failures should be close to 0.5. As published earlier, Ti/Tv ratio for whole genome falls between 2.05 - 2.15 for both known and unknown SNPs. SInC incorporates a user-defined Ti/Tv ratio for simulation of SNVs.

All the scripts to simulate variants and generate reads used default parameters and details of the scripts used are given in the Additional file 5.

## Results and discussion

We have developed a simulator for all commonly occurring biological variants in the genome along with a read generator. We compared the latest pick of simulators with SInC simulator and read generator. In our model for SNV simulation, we have limited the range of simulation of SNVs using a distribution of distance between two consecutive SNVs. Based on SNV frequency studies in human genome [31,34], under default simulation parameters the mean distance between two consecutive SNVs, DAvg, is set dynamically between 300 to 1000 bases depending upon user defined input for SNV rate. In indel simulations, the complexity of simulation depends on the frequency of indels in the simulated data. In the default mode for indel simulation, the algorithm is sensitive to the natural frequencies and size ranges as evidenced from existing literature [33,35]. The model for CNV simulation is an extension of the indel simulation, wherein the CNVs are dynamically generated while maintaining the allele specificity and genomic positions of indels simulated in the prior step. The simulated variants are captured in log files, combined with input allele fasta files and processed by a multi-threaded process to enable fast-paced read generation.

In order to assess the number of variants post simulation and read-generation in comparison to the number of variants that a sensitive variant caller like GATK [4] identifies, we used variant re-discovery rate as one of the parameters of evaluation. Variant re-discovery, although not linked with the efficiency of the simulator, can be used as one of the parameters to judge the combined efficiency of the simulator + read-generator + variant calling process. The process of re-discovery can be impacted by the read-generation step, which incorporates different error models and/or base quality values. Given the fact that we used the same tool (GATK [4] for SNPs and Pindel [6] for indels) across all the simulators + read-generator pairs, discrepancies in the number of variants rediscovered provided us with indirect evidence on the individual tools’ efficiency. The most likely explanation for varying re-discovery rate could be due to different methodology adopted during the process of read generation where different error models (and quality values) are taken into consideration. Additionally, variant re-discovery rate is a widely used parameter to assess the quality of variant calling and analysis, including in the 1000 genome project. In order to delineate coverage from that of combined simulation + read-generation + variant calling and re-discovery, we simulated reads at 10X, 20X or 30X coverage and found that the coverage does not affect the re-discovery rate of SNPs using SInC (Additional file 6). The SNV rediscovery percentage suggested that SInC was at par with pIRS in the efficiency of simulating SNVs and comprehensively outperformed both GEMsim and dwgSIM (Figure 2A), suggesting the role of similar error-profile based model during the read generation process. Although other tools like dwgSIM and GEMsim are close to SInC in homozygous rediscovery rate (Figure 2D), SInC outperforms both these tools for heterozygous rediscovery rate (Figure 2C) suggesting the importance in simulating both homozygous and heterozygous real variants. In the rediscovery of indels, SInC emerges as the only simulator with the highest percentage of total rediscovered indels, ahead at least by 15% from the closest contender pIRS (Figure 2B). We further tested the accuracy of the rediscovered indels by adding a size-based constraint and estimated the percentage rediscovered in the size ranges containing 1 to 6, 7 to 10, 11 to 20 and 21 to 100 nucleotide long indels. These size ranges were simulated due to their overall high (greater than 95%) natural prevalence in human genomes [35]. This exercise corroborated the superiority of SInC in detecting indels while retaining the size-based constraints implicated in the simulation algorithm in comparison to the other tools tested (Figure 2B). The numbers of SNVs and indels rediscovered by SInC are especially important because the total number of SNVs simulated by SInC is about 10-20% more than the other tools tested and 20-40% higher for indels. Another significant advantage of SInC is apparent from the rediscovered heterozygous SNVs. As depicted in Figure 2C, the difference in homozygous SNV rediscovery is rather conserved across the simulators compared to Figure 2D, which gives SInC an edge in conservation of zygosity of the calls post read generation. Notably, pIRS although uses a similar error-profile as SInC, it does not catalog the simulated SNVs to facilitate rediscovery of heterozygous and homozygous SNVs separately. The CNV module of SInC simulator was used in a previous study to test a CNV prediction tool, COPS [17], and was used to compare its accuracy and sensitivity to other popular CNV prediction tools. We were unable to perform a comparative analysis of the CNV module in SInC due to the unavailability of any published tools that can simulate CNVs. However, as previously shown [17], the percentage rediscovery using multiple CNV discovery tools like CNAseg CNV-seq, CNVnator and SVDetect yielded >90% CNVs.

Next, we wanted to test the speed of SInC read generator. Figure 3 depicts the advantages that SInC provides during read generation due to implementation of a “divide and conquer” approach by efficient utilization of C thread functions. The tool was tested for its processing capability under a range of multi-threaded options ranging from default utilization of 1 core to a maximum utilization of 4 cores. SInC accomplished read generation at least one and a half times faster than pIRS and three times faster than ART; the two most recent Illumina read simulators (see Additional file 2). The time profile demonstrated substantial reduction in time footprint using SInC in comparison to the other tools sampled in our study. This difference in generation time of simulated data is reflected clearly in generating high coverage datasets from large genomes, human genome in our case as shown in Figures 3B and C.

Although there are a multitude of popular tools capable of predicting genomic variations using high-throughput sequence data, the generality of such tools are questionable. In many ways, a simulated dataset is crucial towards determining the success of predictive algorithms in the context of real dataset. Simulators that can simulate variants and generate reads are valuable tools used for developing and testing tools for sequence data analysis. An ideal tool that can both simulate multiple variant types (SNVs, indels and CNVs) and generate sequencing reads taking into account a realistic platform-specific quality-profile of an sequencing instrument is currently lacking. We tried filling this void by designing a versatile and fast tool that can generate multiple types of biological variants (SNVs, indels and CNVs) and can run on a minimalistic quad core desktop computer using multi-threaded option. The time advantage obtained in SInC could be attributed to the optimized algorithms and efficient use of C thread functions to manage the I/O streams. This advantage is also obvious in a single core, which delegates the bulk of the data generation to multiple threads to ensure efficient use of memory in line with “divide and conquer” approach. The optimization of multiple core usage is available upto 4 cores in quad-core architecture.

Another major functional advantage of this tool is its ability to simulate CNVs. CNVs have been shown to contribute more towards genetic diversity than SNVs and are conspicuous by their pervasiveness in human genome [36-39]. The advent of NGS platforms has geared multiple efforts to build frameworks towards identifying CNVs and assess their penetrance in disease etiology. However, most of these efforts are only partially effective in capturing population-based generalizations. In order to build a robust and generic framework, it is imperative to build exhaustive datasets with the known signatures and explore the range of false discovery rates inherent to the tools and subsequently improve them. The ability to create such datasets will definitely improve the approach and accuracy of predictions made by existing tools. Hence, a flexible, user-input based simulator has substantial application in building useful datasets allowing for improvement of current approaches towards variant discovery as a whole. Although there have been efforts in the past to discovering CNVs using NGS data, currently there are no available simulators to fine-tune CNV detection algorithms. SInC simulator not only fulfills the simulation of CNVs but an additional functionality of SInC simulator is to generate allele-specific CNVs. This is particularly useful if one has to understand the copy number changes at an allelic level important for many diseases [40,41].

Production of large amount of heterogeneous data in high-throughput biology requires sophisticated computational tools for efficient analysis, storage, sharing and archiving. This requires resources, both software and hardware, and interoperability of computational resources. A common practice among computational biologist is to use simulated data to test the efficacy of the tools before applying them to real dataset. Although there are many simulators available currently, there is none that suits the need of every computational biologist wanting to make tools for short-read sequence data. Keeping this in mind, we have developed a tool to help computational biologists create simulated datasets using only one simulator that can span across sequencing platforms and variant types (SNVs, indels and CNVs). Although, SInC simulator was tested with human genome, it is versatile to address the complexity of any genome, its substitution rate, variant frequency and transition to transversion ratio. Large genomes, like that from many plants, need time to generate simulated reads at high coverage and this is where the multi-threaded capability of SInC scores high in comparison to other tools. By using a standalone quality-score distribution model of real dataset, SInC provides an opportunity to individual user to generate reads at different read lengths but with realistic quality.

## Conclusions

We report a tool called SInC that can simulate and generate short sequence reads with different types of biological variants. The ability of SInC to generate realistic fastq reads based on Illumina read quality profiles along with its capacity to simulate multiple biological variants and generate reads concurrently makes it a powerful option in a variety of simulation studies and a part of computational biologists’ essential toolkit.

## Availability and requirements

**Project name:** sincsimulator

**Project home page:**http://sourceforge.net/projects/sincsimulator

**Operating system(s):** Linux

**Programming Language:** C

**Other Requirements:** GNU Scientific Library(gsl library), pthreads library

**License:** Creative Commons Attribution Non-Commercial License V2.0

**Any restrictions to use by non-academics**: License needed

## Abbreviations

SNP: Single nucleotide polymorphism; Indel: Insertions and deletions; CNV: Copy number variations.

## Competing interests

Both SP and BP are paid by Strand Life Sciences. The authors declare that they have no other competing interests.

## Authors’ contributions

BP conceived the project and come up with the tool’s parameters. SP designed the analytical workflow and fixed bugs in the code. AAR coded the first version of the tool. SG helped in filling parts of the code, fixed bugs and tested the tool. SP and BP wrote the manuscript. All authors read and approved the manuscript.

## Supplementary Material

Additional file 1 **SInC SNP distribution.** A) A Gaussian distribution was implemented to dynamically allocate distance between two SNVs. Under default conditions, which follows a SNV rate of 0.001, the mean distance, D_Avg_, between two SNVs was set to 1000 as evidenced by studies from 1000 genome project. Also, a lower limit of D_Avg_ was set to 300 based on these studies allowing us to dynamically simulate SNVs of biological relevance. B) Normalized frequency distribution of simulated SNVs per chromosome in hg19 assembly. Click here for file

Additional file 2 **Time profiles of SInC, and variant re-discovery numbers.** Time elapsed to perform one complete simulation with default options using 1–4 cores A) For chromosome 22 at 15X B) For human whole genome (hg19) at 5X. SNPs were re-discovered using GATK and indels with Pindel. Click here for file

Additional file 3 **SInC indel distribution.** A) The size based frequency distribution of indels used in SINC based on literature evidence from Millis et al. B) Normalized frequency distribution of simulated indels per chromosome in hg19 assembly. Click here for file

Additional file 4 **Illumina-derived base quality score distribution used to generate reads by SInC.** Quality score distribution of reads from training sets vs reads simulated using SInC; A) for forward read B) for reverse reads. Top panel: training set, bottom panel: reads simulated using SInC. Click here for file

Additional file 5 Scripts used to run various tools. Click here for file

Additional file 6 **Coverage verses SNP re-discovery rate.** Effect of coverage on combined process of simulation + read-generation + variant calling and re-discovery. Click here for file
