# Supplementary figures and images for: SInC: an accurate and fast error-model based simulator for SNPs, Indels and CNVs coupled with a read generator for short-read sequence data
Source: BMC Bioinformatics. 2014 Feb 5;15:40. doi: 10.1186/1471-2105-15-40 (PMC3926339; doi:10.1186/1471-2105-15-40)

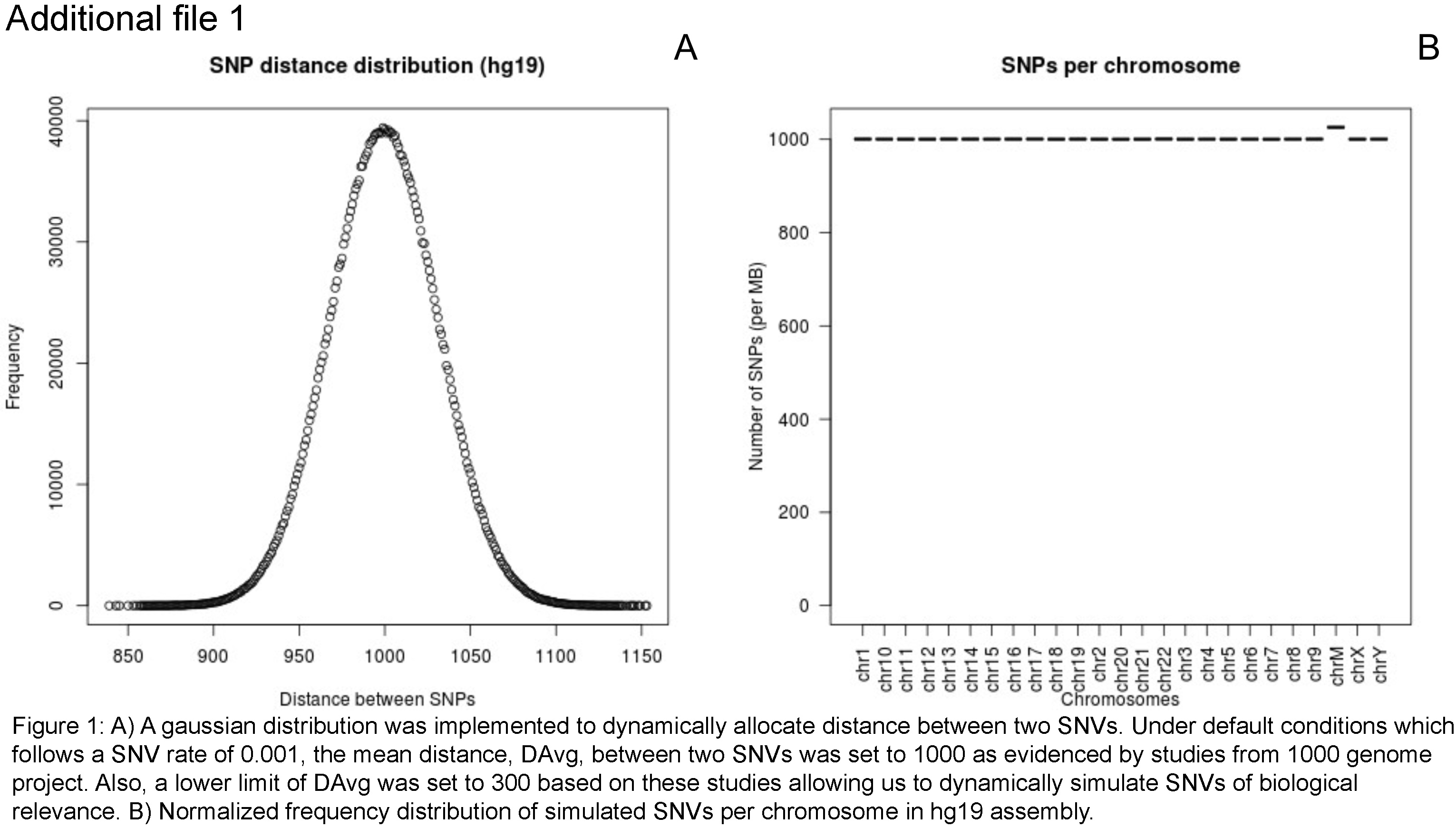

Supplement: Additional file 1 — SInC SNP distribution. A) A Gaussian distribution was implemented to dynamically allocate distance between two SNVs. Under default conditions, which follows a SNV rate of 0.001, the mean distance, DAvg, between two SNVs was set to 1000 as evidenced by studies from 1000 genome project. Also, a lower limit of DAvg was set to 300 based on these studies allowing us to dynamically simulate SNVs of biological relevance. B) Normalized frequency distribution of simulated SNVs per chromosome in hg19 assembly. [file 1471-2105-15-40-S1.jpeg]

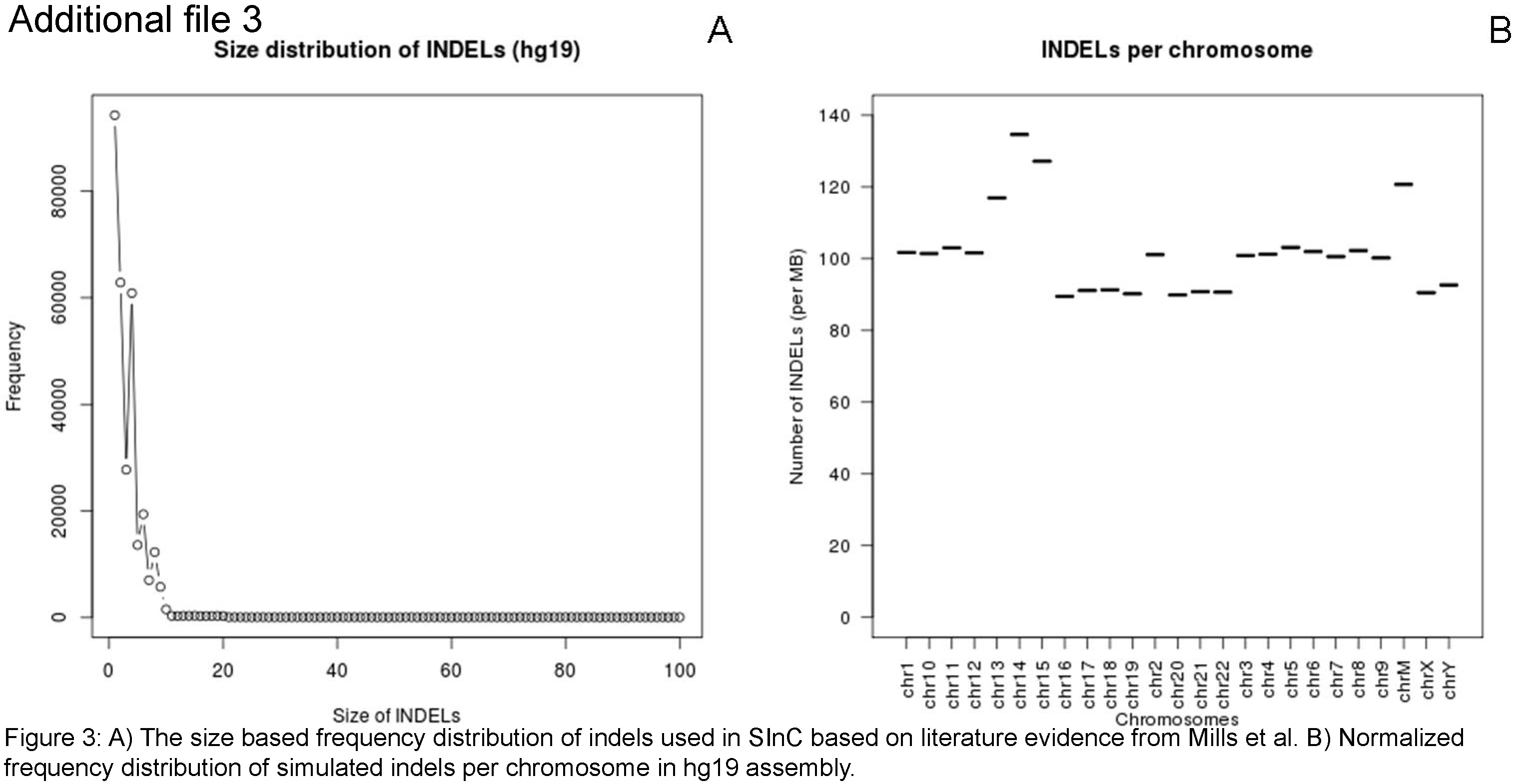

Supplement: Additional file 3 — SInC indel distribution. A) The size based frequency distribution of indels used in SINC based on literature evidence from Millis et al. B) Normalized frequency distribution of simulated indels per chromosome in hg19 assembly. [file 1471-2105-15-40-S3.jpeg]

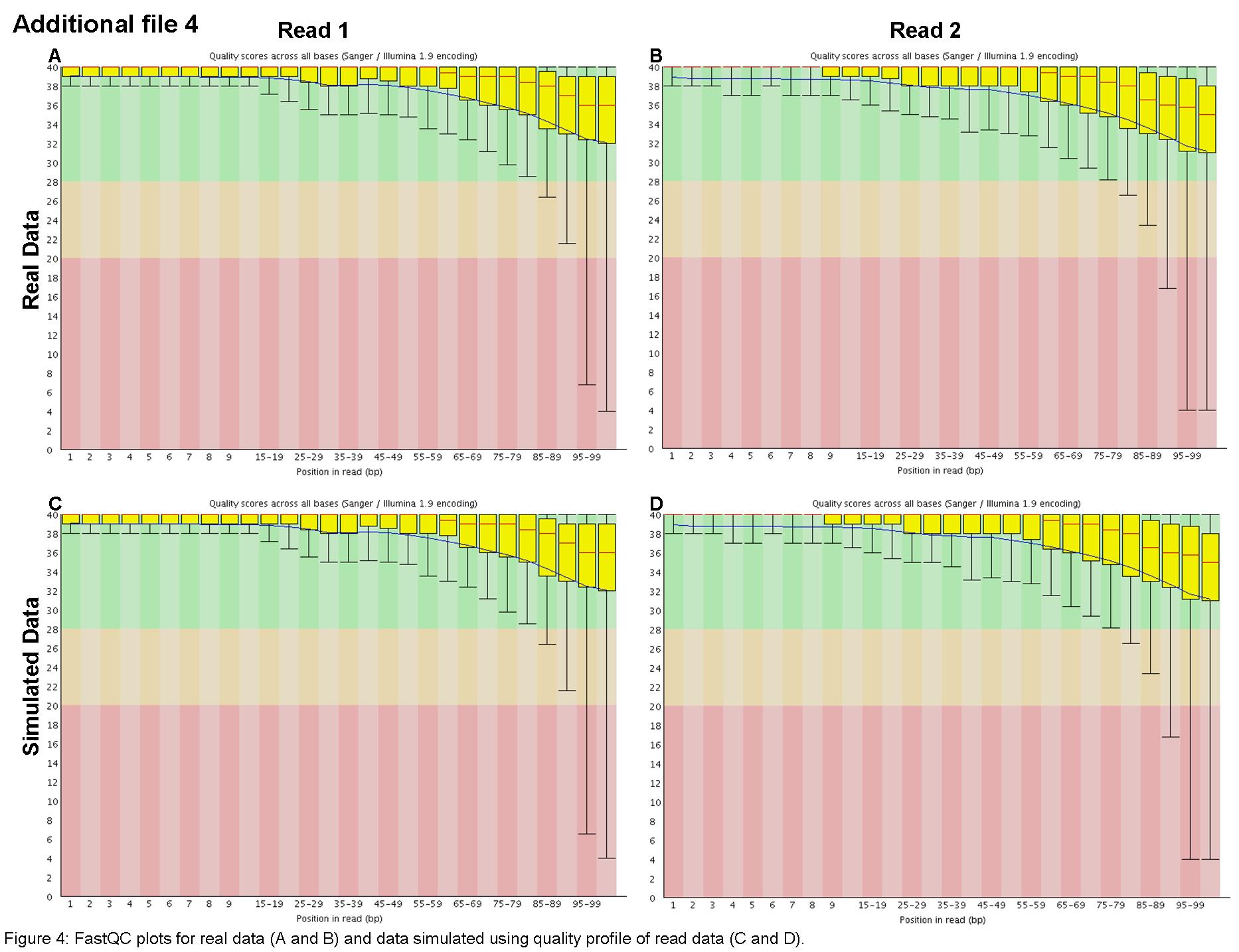

Supplement: Additional file 4 — Illumina-derived base quality score distribution used to generate reads by SInC. Quality score distribution of reads from training sets vs reads simulated using SInC; A) for forward read B) for reverse reads. Top panel: training set, bottom panel: reads simulated using SInC. [file 1471-2105-15-40-S4.jpeg]

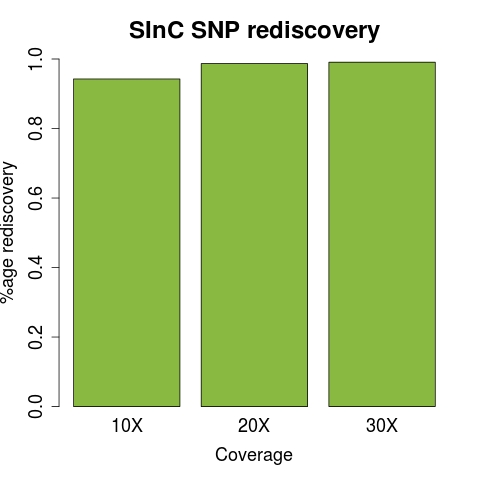

Supplement: Additional file 6 — Coverage verses SNP re-discovery rate. Effect of coverage on combined process of simulation + read-generation + variant calling and re-discovery. [file 1471-2105-15-40-S6.jpeg]
