# Supplementary material for: SInC: an accurate and fast error-model based simulator for SNPs, Indels and CNVs coupled with a read generator for short-read sequence data
Source: BMC Bioinformatics. 2014 Feb 5;15:40. doi: 10.1186/1471-2105-15-40 (PMC3926339; doi:10.1186/1471-2105-15-40)
Supplement: Additional file 2 — Time profiles of SInC, and variant re-discovery numbers. Time elapsed to perform one complete simulation with default options using 1–4 cores A) For chromosome 22 at 15X B) For human whole genome (hg19) at 5X. SNPs were re-discovered using GATK and indels with Pindel. [file 1471-2105-15-40-S2.doc]

**Additional file 2:** Time profiles of SInC, and variant re-discovery numbers. Time elapsed to perform one complete simulation with default options using 1-4 cores A) For chromosome 22 at 15X B) For human whole genome (hg19) at 5X. SNPs were re-discovered using GATK and indels with Pindel.
**Time profile**

| Tool | Version | # cores | chr22 | hg19 |
| --- | --- | --- | --- | --- |
| pirs | 1.00 | 1 | 225 | 5775 |
| dwgsim | 0.1.10 | 1 | 69 | 15993 |
| Gemsim | 1.6 | 1 | 26778 | 401640 |
| art | banana_packages | 1 | 130 | 11718 |
| SInC | 1.00 | 1 | 123 | 3762 |
| SInC | 1.00 | 2 | 86 | 3124 |
| SInC | 1.00 | 3 | 84 | 3150 |
| SInC | 1.00 | 4 | 94 | 3285 |

**SNP re-discovery**

| Tool | coverage | # SNPs simulated | %redisc PASS | %het PASS redisc | %hom PASS redisc |
| --- | --- | --- | --- | --- | --- |
| pirs | 20.56 | 35150 | 97.19 | NA | NA |
| dwgsim | 19.46 | 31291 | 86.53 | 80.72 | 97.44 |
| gemsim | 21.75 | 23822 | 84.68 | NA | NA |
| SInC | 19.48 | 34914 | 98.68 | 97.29 | 99.00 |

**Indel re-discovery**

| Tool | coverage | INDEL size | # INDELs simulated | % INDELs redisc |
| --- | --- | --- | --- | --- |
| pirs | 22.03 | all | 42877 | 75.64 |
|  |  | 1-6 | 42848 | 75.77 |
|  |  | 7-10 | 0 | 0.00 |
|  |  | 11-20 | 0 | 0.00 |
|  |  | 20- | 29 | 37.93 |
| dwgsim | 19.46 | all | 42388 | 44.03 |
|  |  | 1-6 | 42375 | 57.04 |
|  |  | 7-10 | 13 | 23.08 |
|  |  | 11-20 | 0 | 0.00 |
|  |  | 20- | 0 | 0.00 |
| SInC | 19.51 | all | 50371 | 95.28 |
|  |  | 1-6 | 44577 | 95.49 |
|  |  | 7-10 | 4248 | 94.33 |
|  |  | 11-20 | 526 | 95.44 |
|  |  | 20- | 1020 | 72.65 |
|  |  | 21-50 | 417 | 91.13 |
