# Supplementary material for: SInC: an accurate and fast error-model based simulator for SNPs, Indels and CNVs coupled with a read generator for short-read sequence data
Source: BMC Bioinformatics. 2014 Feb 5;15:40. doi: 10.1186/1471-2105-15-40 (PMC3926339; doi:10.1186/1471-2105-15-40)
Supplement: Additional file 5 — Scripts used to run various tools. [file 1471-2105-15-40-S5.pdf]

Additional file 5

==> Scripts time Profile <==
pIRS(1.00):

-> pirs diploid -i chr22.fa -s 0.001 -c 0 -o chr22_pirs_S0.001
-> pirs simulate -i chr22.fa -I chr22_pirs_S0.001.snp.indel.inversion.fa -x 10 -m 300 -v 50 -g 0 -Q 33 -c 0 -o chr22_pirs_S0.001_15X

-> pirs diploid -i hg19.fa -c 0 -o hg19_pirs_timeProfile
-> pirs simulate -i hg19.fa -I hg19_pirs_S0.001.snp.indel.inversion.fa -x 10 -m 300 -v 50 -g 0 -Q 33 -c 0 -o hg19_pirs_S0.001_15X

-> Note: value for -x is 10 for 15X coverage because pirs calculates the number of reads including the N regions.

dwgsim(0.1.10):

-> dwgsim -d 300 -N 2600000 -1 100 -2 100 chr22.fa chr22_dwgsim_S0.001_2.6M

-> dwgsim -d 300 -N 70000000 -1 100 -2 100 hg19.fa hg19_dwgsim_S0.001_70M

Gemsim(1.6):

-> GemHaps.py -r chr22.fa -g '.80,28000 .20,7000'
-> GemReads.py -p -r chr22.fa -n 2600000 -g chr22.txt -l 100 -m models/ill100v5_p.gzip -q 33 -u 300 -s 50 -o chr22_gemsim_S0.001_2.6M

-> GemHaps.py -r hg19.fa -g '.80,28000 .20,7000'
-> GemReads.py -p -r hg19.fa -n 70000000 -g hg19.txt -l 100 -m models/ill100v5_p.gzip -q 33 -u 300 -s 50 -o hg19_gemsim_S0.001_70M

SInC(1.00):

-> SInC_simulate -p 0 chr22.fa
-> SInC_readGen -C 7.5 -T 1 -R 100 chr22.fa_allele_1_S_0.0010_I_0.0001_C_0.00_1000_150000.fa 100_bp_read_1_profile.txt 100_bp_read_2_profile.txt
-> SInC_readGen -C 7.5 -T 1 -R 100 chr22.fa_allele_2_S_0.0010_I_0.0001_C_0.00_1000_150000.fa 100_bp_read_1_profile.txt 100_bp_read_2_profile.txt

-> SInC_readGen -C 7.5 -T 2 -R 100 chr22.fa_allele_1_S_0.0010_I_0.0001_C_0.00_1000_150000.fa 100_bp_read_1_profile.txt 100_bp_read_2_profile.txt
-> SInC_readGen -C 7.5 -T 2 -R 100 chr22.fa_allele_2_S_0.0010_I_0.0001_C_0.00_1000_150000.fa 100_bp_read_1_profile.txt 100_bp_read_2_profile.txt

-> SInC_readGen -C 7.5 -T 3 -R 100 chr22.fa_allele_1_S_0.0010_I_0.0001_C_0.00_1000_150000.fa 100_bp_read_1_profile.txt 100_bp_read_2_profile.txt

-> SInC_readGen -C 7.5 -T 3 -R 100
chr22.fa_allele_2_S_0.0010_I_0.0001_C_0.00_1000_150000.fa 100_bp_read_1_profile.txt
100_bp_read_2_profile.txt

-> SInC_readGen -C 7.5 -T 4 -R 100
chr22.fa_allele_1_S_0.0010_I_0.0001_C_0.00_1000_150000.fa 100_bp_read_1_profile.txt
100_bp_read_2_profile.txt
-> SInC_readGen -C 7.5 -T 4 -R 100
chr22.fa_allele_2_S_0.0010_I_0.0001_C_0.00_1000_150000.fa 100_bp_read_1_profile.txt
100_bp_read_2_profile.txt

-> SInC_simulate -p 0 hg19.fa
-> SInC_readGen -C 7.5 -T 1 -R 100
hg19.fa_allele_1_S_0.0010_I_0.0001_C_0.00_1000_150000.fa 100_bp_read_1_profile.txt
100_bp_read_2_profile.txt
-> SInC_readGen -C 7.5 -T 1 -R 100
hg19.fa_allele_2_S_0.0010_I_0.0001_C_0.00_1000_150000.fa 100_bp_read_1_profile.txt
100_bp_read_2_profile.txt

-> SInC_readGen -C 7.5 -T 2 -R 100
hg19.fa_allele_1_S_0.0010_I_0.0001_C_0.00_1000_150000.fa 100_bp_read_1_profile.txt
100_bp_read_2_profile.txt
-> SInC_readGen -C 7.5 -T 2 -R 100
hg19.fa_allele_2_S_0.0010_I_0.0001_C_0.00_1000_150000.fa 100_bp_read_1_profile.txt
100_bp_read_2_profile.txt

-> SInC_readGen -C 7.5 -T 3 -R 100
hg19.fa_allele_1_S_0.0010_I_0.0001_C_0.00_1000_150000.fa 100_bp_read_1_profile.txt
100_bp_read_2_profile.txt
-> SInC_readGen -C 7.5 -T 3 -R 100
hg19.fa_allele_2_S_0.0010_I_0.0001_C_0.00_1000_150000.fa 100_bp_read_1_profile.txt
100_bp_read_2_profile.txt

-> SInC_readGen -C 7.5 -T 4 -R 100
hg19.fa_allele_1_S_0.0010_I_0.0001_C_0.00_1000_150000.fa 100_bp_read_1_profile.txt
100_bp_read_2_profile.txt
-> SInC_readGen -C 7.5 -T 4 -R 100
hg19.fa_allele_2_S_0.0010_I_0.0001_C_0.00_1000_150000.fa 100_bp_read_1_profile.txt
100_bp_read_2_profile.txt

==> Scripts rediscovery SNPs <==
dwgsim(0.1.10):

-> dwgsim -r 0.001 -N 3400000 -1 100 -2 100 -q I chr22.fa chr22_100L_3.4M_0.001S

pIRS(1.00):

-> pirs diploid -i chr22.fa -s 0.001 -o chr22.fa_S0.001 >SimDiploid.out 2>SimDiploid.err
-> gunzip chr22.fa_S0.001.snp.indel.invertion.fa.gz
-> pirs simulate -i chr22.fa -I chr22.fa_S0.001.snp.indel.invertion.fa -s humNew.PE100.matrix.gz -l 100 -x 15 -a 0 -g 0 -M 0 -Q 33 -c 0 -o chr22.fa_15X_100L_S0.001_SNPs

Gemsim(1.6):

-> GemHaps.py -r chr22.fa -g '.80,28000 .20,7000'
-> GemReads.py -p -r chr22.fa -n 3800000 -g chr22.txt -l 100 -m models/ill100v5_p.gzip -q 33 -u 300 -s 50 -o chr22_gemsim_S0.001_3.8M

SInC(1.00):

-> SInC_simulate -S 0.001 -p 0 chr22.fa
-> SInC_readGen -C 7.5 -T 1 -R 100 chr22.fa_allele_1_S_0.0010_I_0.0001_C_0.00_1000_150000.fa 100_bp_read_1_profile.txt 100_bp_read_2_profile.txt
-> SInC_readGen -C 7.5 -T 1 -R 100 chr22.fa_allele_2_S_0.0010_I_0.0001_C_0.00_1000_150000.fa 100_bp_read_1_profile.txt 100_bp_read_2_profile.txt

==> Scripts rediscovery INDELs <==
dwgsim(0.1.10):

-> dwgsim -r 0.001 -R 1.0 -N 3400000 -1 100 -2 100 chr22.fa chr22_I0.001_100L_3.4M_indels

pIRS(1.00):

-> pirs diploid -i chr22.fa -d 0.001 -o chr22.fa_D0.001 >Sim_D0.001_Diploid.out 2>Sim_D0.001_Diploid.err
-> gunzip chr22.fa_D0.001.snp.indel.invertion.fa.gz
-> pirs simulate -i chr22.fa -I chr22.fa_D0.001.snp.indel.invertion.fa -s humNew.PE100.matrix.gz -l 100 -x 15 -a 0 -g 0 -M 0 -Q 33 -c 0 -o chr22.fa_15X_100L_D0.001

SInC(1.00):

-> SInC_simulate -S 0.001 -p 0 chr22.fa
-> SInC_readGen -C 7.5 -T 1 -R 100 chr22.fa_allele_1_S_0.0010_I_0.0001_C_0.00_1000_150000.fa 100_bp_read_1_profile.txt 100_bp_read_2_profile.txt
-> SInC_readGen -C 7.5 -T 1 -R 100 chr22.fa_allele_1_S_0.0010_I_0.0001_C_0.00_1000_150000.fa 100_bp_read_1_profile.txt 100_bp_read_2_profile.txt

==> Novoalign script <==
mapping was done using novoalignMPI(version 02.07.11)
below is the command and parameter used for all the mapping

/Apps/mvapich2/bin/mpiexec.hydra -np $NSLOTS -hostfile $TMPDIR/machines /Apps/NOVO/novoalignMPI/novoalignMPI -d chr22.nidx -f <R1.fastq> <R2.fastq> -a -o SAM -i <insert size>,<standard deviation> '@RG\tID:default\tPU:illumina\tSM:default' > output.sam
samtools view -bS output.sam > output.bam
samtools sort output.bam output.sorted
samtools index output.sorted.bam

==> Pindel script <==

```
# the following script was used(for all samples) to run pindel to detect indels for rediscovery

if [ $# -lt 3 ]
then
	echo -e "Usage:\n\tbash $0 <bam_file> <outfile_id> <output directory>";
	exit 0
fi

export LD_LIBRARY_PATH=$LD_LIBRARY_PATH:/Apps/gcc/gcc-4.5.0/lib64
export BAM_2_PINDEL_ADAPT=/Apps/Pindel/Adaptor_mod.pm
mkdir $3

ln -s $1 ./$2.bam

samtools sort -n $2.bam $2.nSorted
perl /Apps/Pindel/bam2pindel_22sept2010_documented.pl -i $2.nSorted.bam -o $3/$2 -pr novo -pi
200 -om -s $2
cat $3/$2_chr*.txt > $3/$2.txt
/Apps/Pindel/pindel_x86_64 -f chr22.fa -p $3/$2.txt -o $3/$2 -c ALL -T 6
```

==> GATK(V1.2.62) script <==

```
# the following script was used(for all samples) to run GATK to detect SNPs for rediscovery

if [ $# -lt 5 ]
then
	echo -e "Usage:\n\tbash $0 <input bam> <prefix> <RG_tag> <node> <output_dir>"
	exit 1
fi

input=$1
prefix=$2
RG=$3
node=$4
out_dir=$5

mkdir $out_dir

echo "#!/bin/bash" > gatk.$prefix.sh
echo "#$ -N gatk" >> gatk.$prefix.sh
echo "#$ -cwd" >> gatk.$prefix.sh
echo "#$ -V" >> gatk.$prefix.sh
echo "#$ -o $out_dir/\$JOB_ID.\$JOB_NAME.OUT" >> gatk.$prefix.sh
echo "#$ -e $out_dir/\$JOB_ID.\$JOB_NAME.ERR" >> gatk.$prefix.sh
echo "#$ -q all.q@compute-0-$node" >> gatk.$prefix.sh
echo "#$ -pe orte 8" >> gatk.$prefix.sh
echo "#$ -j y" >> gatk.$prefix.sh
echo "#$ -m aes" >> gatk.$prefix.sh
echo "#$ -M saurabh@ganitlabs.in" >> gatk.$prefix.sh
```

```
echo "echo \"$1\" >> $out_dir/gatk_prefix_log; date >> $out_dir/gatk_prefix_log" >>
gatk.$prefix.sh

echo  >> gatk.$prefix.sh
echo "java -Xmx1g -jar /Apps/GATK/GenomeAnalysisTK-1.2-62/GenomeAnalysisTK.jar -I $input
-T RealignerTargetCreator -R chr22.fa -o $out_dir/$prefix.IndelRealigner.intervals --known
1000G_biallelic.indels.hg19.vcf" >> gatk.$prefix.sh
echo  >> gatk.$prefix.sh
echo "java -Xmx4g -jar /Apps/GATK/GenomeAnalysisTK-1.2-62/GenomeAnalysisTK.jar -I $input
-R chr22.fa -T IndelRealigner  --targetIntervals $out_dir/$prefix.IndelRealigner.intervals -o
$out_dir/$prefix.realigned.bam" >> gatk.$prefix.sh
echo  >> gatk.$prefix.sh
echo "java -jar /Apps/picard-tools-1.39/MarkDuplicates.jar INPUT=$out_dir/$prefix.realigned.bam
OUTPUT=$out_dir/$prefix.realigned.DupRem.bam REMOVE_DUPLICATES=true
ASSUME_SORTED=true CREATE_INDEX=true READ_NAME_REGEX='[-a-zA-Z0-9]+:[0-
9]:([0-9]+):([0-9]+):([0-9]+).*' METRICS_FILE=$out_dir/$prefix.picardMetrics.txt" >>
gatk.$prefix.sh
echo  >> gatk.$prefix.sh
echo "samtools index $out_dir/$prefix.realigned.DupRem.bam" >>  gatk.$prefix.sh
echo  >> gatk.$prefix.sh
echo "java -jar /Apps/GATK/GenomeAnalysisTK-1.2-62/GenomeAnalysisTK.jar -R  chr22.fa -
knownSites:VCF dbsnp_132.hg19.vcf -I $out_dir/$prefix.realigned.DupRem.bam -T
CountCovariates -nt 8 -cov QualityScoreCovariate -cov CycleCovariate -recalFile
$out_dir/$prefix.realigned.DupRem_firstRecal.csv --default_read_group $RG --default_platform
illumina" >> gatk.$prefix.sh
echo  >> gatk.$prefix.sh
echo "java -jar /Apps/GATK/GenomeAnalysisTK-1.2-62/GenomeAnalysisTK.jar -R  chr22.fa -I
$out_dir/$prefix.realigned.DupRem.bam -T TableRecalibration -o
$out_dir/$prefix.realigned.DupRem.firstRecal.bam -recalFile
$out_dir/$prefix.realigned.DupRem_firstRecal.csv --default_read_group $RG --default_platform
illumina" >> gatk.$prefix.sh
echo  >> gatk.$prefix.sh
echo "java -jar /Apps/GATK/GenomeAnalysisTK-1.2-62/GenomeAnalysisTK.jar -R  chr22.fa -
knownSites:VCF dbsnp_132.hg19.vcf -I $out_dir/$prefix.realigned.DupRem.firstRecal.bam -T
CountCovariates -nt 8 -cov QualityScoreCovariate -cov CycleCovariate -recalFile
$out_dir/$prefix.realigned.DupRem_secondRecal.csv --default_read_group $RG --default_platform
illumina" >> gatk.$prefix.sh
echo  >> gatk.$prefix.sh
echo "java -Xmx4g -jar /Apps/GATK/GenomeAnalysisTK-1.2-62/AnalyzeCovariates.jar -recalFile
$out_dir/$prefix.realigned.DupRem_secondRecal.csv -outputDir
$out_dir/$prefix.AnalysisCovariateOutput -ignoreQ 5 -resources
/Apps/GATK/GenomeAnalysisTK-1.2-62/resources/" >> gatk.$prefix.sh
echo >> gatk.$prefix.sh
echo "samtools index $out_dir/$prefix.realigned.DupRem.firstRecal.bam" >> gatk.$prefix.sh
echo  >> gatk.$prefix.sh
echo "java -jar /Apps/GATK/GenomeAnalysisTK-1.2-62/GenomeAnalysisTK.jar -R chr22.fa -T
UnifiedGenotyper -I $out_dir/$prefix.realigned.DupRem.firstRecal.bam -nt 8 --dbsnp
dbsnp_132.hg19.vcf -o $out_dir/$prefix.raw.vcf -stand_call_conf 50.0 -stand_emit_conf 10.0 -glm
BOTH" >> gatk.$prefix.sh

echo "Run \"qsub gatk.$prefix.sh\" to run GATK."
```
